# Clinical Use of a Real-World Low Carbohydrate Diet Resulting in Reduction of Insulin Dose, Hemoglobin A1c, and Weight

**DOI:** 10.3389/fnut.2021.690855

**Published:** 2021-08-11

**Authors:** Susan Wolver, Kristen Fadel, Ethan Fieger, Zein Aburish, Brennen O'Rourke, Toni-Marie Chandler, Dorian Shimotani, Natasha Clingempeel, Shuchi Jain, Aashish Jain, Puneet Puri

**Affiliations:** ^1^Virginia Commonwealth University, Medical Center, Richmond, VA, United States; ^2^School of Medicine, Virginia Commonwealth University, Richmond, VA, United States; ^3^Virginia Commonwealth University, Richmond, VA, United States; ^4^Independent Researcher, Richmond, VA, United States

**Keywords:** insulin, obesity, diet, diabetes, low-carbohydrate diet

## Abstract

**Introduction:** Type 2 Diabetes Mellitus (T2DM) is increasing in epidemic proportions. In addition to the morbidity and mortality, for those treated with insulin, the physical, psychological, and financial tolls are often greater. Our real-world study evaluated a Low Carbohydrate Diet (LCD) in patients with T2DM on insulin with respect to glycemic control, insulin reduction, and weight loss.

**Materials and Methods:** A prospective cohort study was conducted via an Electronic Medical Record search for patients attending the Virginia Commonwealth University Medical Weight Loss Program from 2014 to 2020 with Type 2 Diabetes Mellitus who initially presented on insulin. Data was extracted for 1 year after enrollment. The weight loss program focuses on a LCD.

**Results:** Of 185 participants, the mean (± SD) age was 56.1 (9.9) years. Seventy percent were female and 63% were black. Eighty-five completed 12 months (45.9%), reduced their median (25–75% interquartile range, IQR) insulin dose from 69 to 0 units (0–18, *p* < 0.0001), HbA1c from 8 to 6.9% (6.2–7.8, *p* < 0.0001), and weight from 116 to 99 kg (85–120, *p* < 001). Eighty six percent who completed 12 months were able to reduce or discontinue insulin, with 70.6% completely discontinuing. Among all participants who completed 3, 6, or 12 months, 97.6% were able to reduce or eliminate insulin use.

**Conclusion:** In patients with T2DM on a LCD, it is possible to reduce and even discontinue insulin use while facilitating weight loss and achieving glycemic control. A Low Carbohydrate Diet should be offered to all patients with diabetes, especially those using insulin.

## Introduction

Type 2 diabetes mellitus (T2DM) has risen to epidemic proportions in the United States (US). The 2020 Centers for Disease Control (CDC) National Diabetes Statistics Report found that 34.2 million Americans have diabetes, with T2DM accounting for 90–95% of cases, and over 88 million American adults have prediabetes (1). These numbers have steadily increased as 1.5 million Americans are diagnosed with diabetes annually, yielding a prevalence of 10.5% in 2018 compared with only 0.93% in 1958 (2).

Type 2 diabetes mellitus is associated with considerable morbidity, often leading to serious micro- and macro-vascular complications. Importantly, 89% of patients with diabetes were also classified as being in the overweight or obese categories (3). Obesity is also a major contributor to chronic health conditions (4), with a prevalence of 42.4% in 2018 (5). Thus, obesity cannot be ignored in the treatment of T2DM.

Lifestyle interventions, such as diet, exercise, and weight loss, are critical to controlling diabetes and preventing progression and are considered first-line treatments; however, success with these interventions is often limited, necessitating medication escalation which often includes insulin. In 2018, 7.5% of individuals with diabetes were on insulin, and this number is expected to increase in the coming years (6).

Though it is clear that insulin helps control serum glucose levels and the progression of microvascular manifestations, there are several drawbacks to insulin treatment that cannot be overlooked. Primarily, people on insulin tend to gain weight, the reason for which is likely multifactorial. Insulin is a known obesogenic medication, and people often increase their carbohydrate consumption in response to a perceived threat or experience of hypoglycemia. Weight gain in diabetes can significantly intensify the cardiac risk profile of a patient (7).

Prior to the use of medication in diabetes, a dietary modification was the only available treatment. Dr. William Osler, the father of internal medicine, recommended carbohydrate restriction for treating diabetes as early as 1923 in his textbook, *Principles and Practice of Medicine* (8); however, dietary guidance for diabetes over the last several decades has been to maintain a certain amount of carbohydrate intake with each meal. In 2019, the CDC recommended that 50% of the calories of a patient come from carbohydrates, amounting to about 200–225 g of carbohydrates per day from an 1,800 Kcal/day diet (9). The prevalence of T2DM has continued to rise despite these guidelines, and due to considerable success, a renewed interest in therapeutic carbohydrate restriction has been mounting. A low carbohydrate ketogenic diet (LCKD) incorporates a marked reduction in carbohydrate intake, usually to <20–50 g per day, to induce metabolic changes that favor fat utilization as the primary energy source *via* ketone formation (10). An LCKD has been shown to improve glycemic control, body weight, and other metabolic parameters, and studies have demonstrated greater weight loss in participants on the LCKD compared with a low-fat diet (11). Thus, the LCKD is a promising treatment option for patients with both T2DM and obesity.

This study sought to evaluate, in a real-world clinical setting, the role of a low carbohydrate diet (LCD) in participants with T2DM on insulin with respect to glycemic control, insulin reduction, and weight loss.

## Materials and Methods

In a prospective cohort study of participants attending the Virginia Commonwealth University Medical Weight Loss Program from 2014 to 2020, an electronic medical record (EMR) search was conducted to identify those with T2DM who initially presented on insulin. Data were extracted for 1 year after enrollment and included 0, 1, 3, 6, and 12-month visits to analyze changes in insulin requirements, weight, laboratory values, and medications. There are no constraints on participation in the program regarding BMI, previous weight loss surgery, or medical comorbidities; however, since the weight loss program operates within a general internal medicine clinic, all participants were adults.

The program focuses on intensive carbohydrate reduction and is led by a general internist, who is a diplomate of the American Board of Obesity Medicine, with assistance from a health coach and health psychologists. Participants initially attend a 90 min class on how to implement an LCKD. They are given a list of foods to eat and avoid which approximates 20 g of total carbohydrates per day from non-starchy vegetables and are told to eat protein to satiety. Natural fats, such as cheese, cream, butter, and oil, are encouraged within limits. The program emphasizes “whole foods,” and recommends avoiding processed food as much as possible. For simplicity and sustainability, participants are not required to count carbohydrates or monitor for ketones. Although most are taught the LCKD, it is likely that not all are in ketosis. Since we did not measure ketones, we will refer to the dietary intervention as an LCD rather than an LCKD.

At the initiation of the LCD, sulfonylureas and sodium-glucose co-transporter-2 inhibitors (SGLT2 inhibitors) are stopped due to the risk of hypoglycemia and ketoacidosis, respectively. Metformin is added or maintained where possible, and in some, injectable glucagon-like peptide 1 agonist (GLP-1) medications are started. Participants are initially required to send daily blood glucose readings through the patient portal of the EMR until they are either off insulin or on a stable dose and home blood glucose readings are controlled.

Exercise is encouraged throughout the program as part of an overall healthy lifestyle but is not specifically part of the initial plan. Participants have access to a Facebook® support group and lifestyle coaching classes, which are a series of four small-group sessions facilitated by a health coach to discuss challenges and to learn behavioral modification for successful dietary adherence. Individuals have monthly visits with a provider to assess for compliance using dietary recall and self-reported adherence. If they are unable to keep their carbohydrates low enough to be in the ketogenic range, they are counseled on trying to reduce carbohydrates intake as much as possible and are given smaller goals. Participants are followed indefinitely; however, the time between visits is lengthened once they become successful and more confident.

Study data were collected and managed using Research Electronic Data Capture (REDCap) electronic data capture tools hosted at Virginia Commonwealth University (12, 13). REDCap is a secure, web-based software platform designed to support data capture for research studies, providing the following services: (1) an intuitive interface for validated data capture, (2) audit trails for tracking data manipulation and export procedures, (3) automated export procedures for seamless data downloads to common statistical packages, and (4) procedures for data integration and interoperability with external sources.

Descriptive analysis with mean and SD was used for normally distributed data, whereas median with interquartile range (IQR) was used for data without normal distribution. Contingency analysis with Pearson's chi-squared test was applied for categorical data. For continuous data, ANOVA with multiple pairwise comparisons was used for normally distributed data, whereas nonparametric multiple comparisons with Wilcoxon test of each pair was used for data without normal distribution. A value of *p* < 0.05 demonstrated statistical significance.

## Results

The EMR data extraction yielded 286 participants; however, 64 were disqualified due to not actually being in the program, not on insulin, or on an insulin pump since daily doses can be hard to quantify. Of the remaining 222, 37 had only one visit and were excluded from the analysis. The mean (± SD) age of the remaining 185 participants, who began the program on insulin, was 56.1 (9.9) years, 70% were female and 63% were black. The mean starting insulin dose was 94 (81) units, hemoglobin A1c (HbA1c) was 8.6% (1.8), weight was 120 (29.7) kg, and BMI was 42.6 (9.6) kg/m^2^ (Table 1). Other than insulin, diabetic medication use at baseline revealed that 97 were on metformin, 40 were on a GLP-1, 18 were on a Dipeptidyl Peptidase-4 inhibitor, 11 were on an SGLT2 inhibitor, and 10 were on a sulfonylurea. In addition to insulin, 1 participant was on 4 agents, 6 were on 3 agents, 36 were on 2 agents, and 19 were on 1 agent.

**Table 1:** Demographic and clinical profile of study population.

	**Total (*N* = 185)**	**12 month completers (*N* = 85)**	**6 month completers (*N* = 47)**	**3 month completers (*N* = 22)**	** <3 month completers (*N* = 31)**	***p*-value**
Age (mean, SD)	56.1 (9.9)	57.1 (9.5)	55.6 (10.2)	55.3 (11.7)	54.6 (9.3)	0.61
Sex (F/M)	130/55 (70%/30%)	57/28	39/8	15/7	19/12	0.15
Race (B/O/W, %)	117/4/64 (63%/2%/35%)	51/2/32	30/1/16	15/0/7	21/1/9	0.96
Weight (kg)	120 (29.7)	121.1 (30.8)	120.6 (31.9)	180.5 (32)	117.8 (21.4)	0.95
BMI	42.6 (9.6)	43.1 (9.7)	43.6 (10.8)	40.6 (10.5)	41.6 (7.2)	0.60
Insurance (Both/Private/ Public/Unknown)	25/78/78/4 (14%/42%/42%/2%)	17/31/34/3	6/20/20/1	1/12/9/0	1/15/15/0	0.32
HTN (No/Yes)	17/168 (9%/91%)	2/83	4/43	6/16	5/26	0.002*
CAD (No/Yes)	158/27 (85%/15%)	70/15	43/4	18/4	27/4	0.51
Hyperlipidemia (No/Yes)	53/132 (29%/71%)	23/62	9/38	10/12	11/20	0.16
Sleep Apnea (No/Yes)	121/64 (65%/35%)	53/32	33/14	17/5	18/13	0.40
Transplant hx (No/Yes)	166/19 (90%/10%)	75/10	43/4	21/1	27/4	0.71
CKD (No/Yes)	142/43 (77%/23%)	63/22	38/9	18/4	23/8	0.75
HIV (No/Yes)	181/4 (98%/2%)	83/2	47/0	22/0	29/2	0.24
Depression (No/Yes)	128/57 (69%/31%)	64/21	36/11	13/9	15/16	0.02*
Previous weight loss surgery (No/Yes)	181/4 (98%/2%)	82/3	46/1	22/0	31/80	0.59

Data shown are mean (SD) in parenthesis; B, black; O, others; and W, white. *statistically significant.

Among the 185 participants, 85 completed 12-month visits, 47 completed 6-month visits, 22 completed 3-month visits, and 31 dropped out prior to 3 months (supp). Comparing the drop-out rate prior to 12 months, there was no difference when comparing Whites to Blacks (*p* = 0.21).

Among the 85 participants that completed 12 months (45.9%), their median insulin dose decreased from 69 to 0 units (0–18, *p* < 0.0001), HbA1c from 8% to 6.9% (6.2–7.8, *p* < 0.0001), and weight from 116 to 99 kg (85–120, *p* < 0.001). Among 12-month completers, 86% were able to reduce or stop insulin, with 70.6% having complete discontinuation. In the 47 participants who completed 6 months, their median insulin dose reduced from 70 to 0 units (0–52, *p* < 0.0001), HbA1c from 8.8% to 7.2% (6.4–8.8, NS), and weight from 116 to 105 kg (89–127, NS). In the 22 participants who completed 3 months, their median insulin dose decreased from 79 to 28 units (0–56, *p* < 0.001), HbA1c from 9.1 to 7.5% (6.8–8.9, *p* < 0.05), and weight from 115 to 109 kg (90–136, NS) (Table 2).

**Table 2:** Weight, HbA1c, and insulin changes during study period for completers.

		**Value type**	**Baseline**	**3 month**	**6 month**	**12 month**
**Insulin (units)** Mean (SD) 94 (81)	12 month completers	Number	*N* = 85	*N* = 82	*N* = 84	*N* = 85
		Min	10	0	0	0
		Max	500	165	170	175
		Median	69	7.5^****^	0^****^	0^****^
		25%	50	0	0	0
		75%	111	31	24	18
		Discontinued				60 (70.6%)
		Reduced				23 (27.1%)
		Increased				2 (2.4%)
		Unchanged				0
	6 month completers	Number	*N* = 47	*N* = 43	*N* = 47	
		Min	10	0	0	
		Max	465	300	300	
		Median	70	0^****^	0^****^	
		25%	40	0	0	
		75%	150	34	52	
		Discontinued			24 (51.1%)	
		Reduced			20 (42.6%)	
		Increased			1 (2.1%)	
		Unchanged			2 (4.3%)	
	3 month completers	Number	*N* = 22	*N* = 22		
		Min	10	0		
		Max	180	180		
		Median	79	28^***^		
		25%	39	0		
		75%	100	56		
		Discontinued		8 (36.4%)		
		Reduced		9 (40.9%)		
		Increased		0		
		Unchanged		5 (22.7%)		
***A1c*****(%)** Mean (SD) 8.6 (1.8)	12 month completers	Number	*N* = 85	*N* = 55	*N* = 59	*N* = 74
		Min	5.6	5.3	5.3	5.1
		Max	14	10.5	11	10.1
		Median	8	7^***^	6.9^***^	6.9^****^
		25%	7.1	6.3	6.2	6.2
		75%	9.3	9.4	8.1	7.8
	6 month completers	Number	*N* = 44	*N* = 24	*N* = 30	
		Min	6.2	5.6	5.3	
		Max	13.9	12.5	12.1	
		Median	8.8	7	7.2	
		25%	7.3	6.4	6.4	
		75%	9.6	9.4	8.8	
	3 month completers	Number	*N* = 20	*N* = 14		
		Min	5.9	6.5		
		Max	14.4	10.8		
		Median	9.1	7.5^*^		
		25%	8.0	6.8		
		75%	11	8.9		
**Weight (kg)** Mean (SD) 120 (29.7)	12 month completers	Number	*N* = 85	*N* = 83	*N* = 84	*N* = 84
		Min	77	69	64	64
		Max	259	259	254	168
		Median	116	108	101^*^	99^***^
		25%	99	96	90	85
		75%	136	127	123	120
	6 month completers	Number	*N* = 47	*N* = 42	*N* = 46	
		Min	69	61	60	
		Max	212	196	201	
		Median	116	112	105	
		25%	97	91	89	
		75%	137	137	127	
	3 month completers	Number	*N* = 22	*N* = 22		
		Min	64	62		
		Max	196	197		
		Median	115	109		
		25%	93	90		
		75%	142	136		

*Compared to baseline*

**** *p < 0.0001*,

*** *p < 0.001*,

* *p < 0.05*.

**Figure 1:**
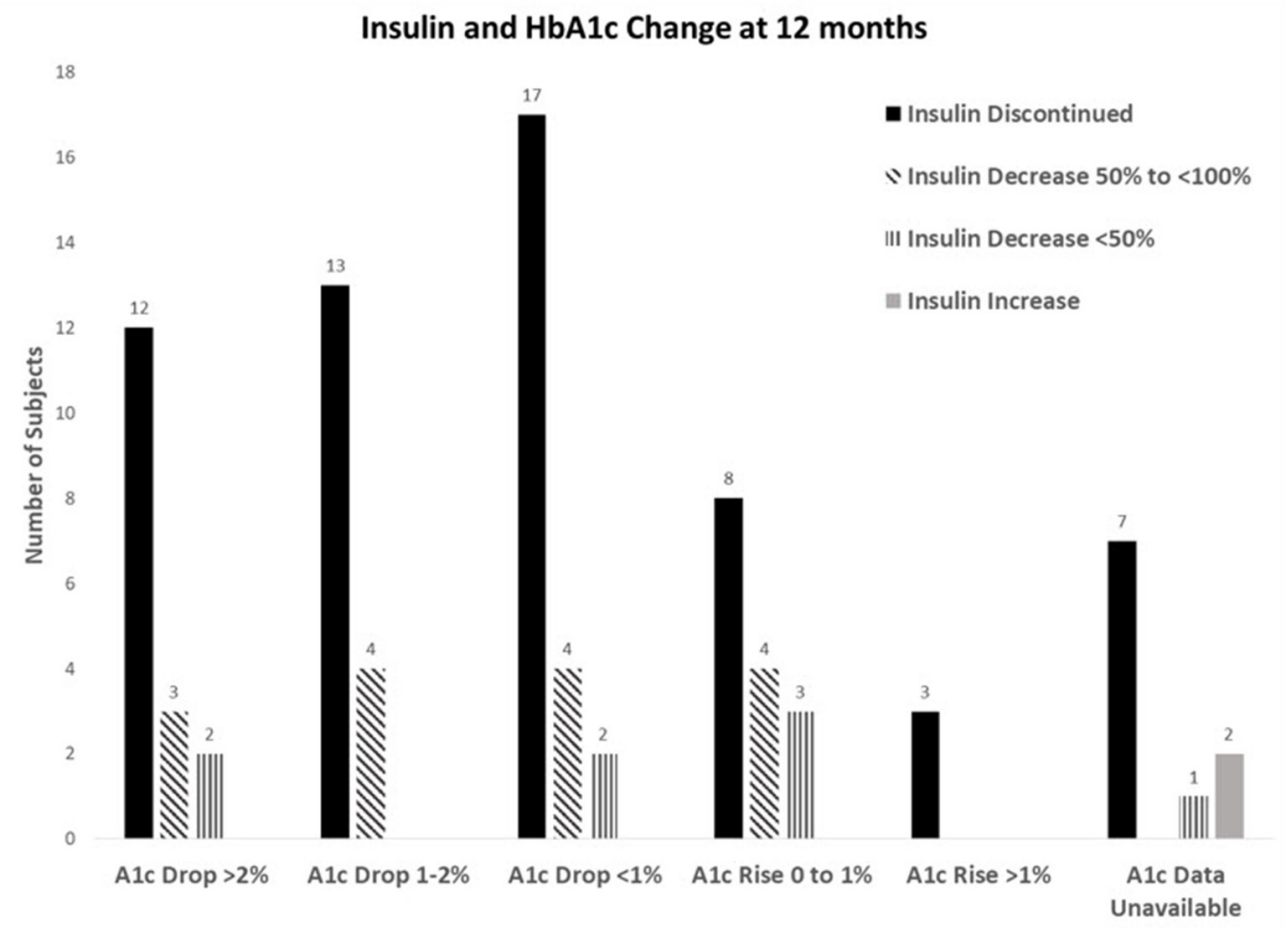
Insulin and HbA1c change at 12 months.

For 12-month completers, 20% had a > 2% reduction in their A1c and of those, 70.6% were also able to stop insulin, and another 29.4% were able to reduce. For those who had an HbA1c reduction of 1–2%, 76.5% were able to stop insulin and 23.5% were able to reduce. For an HbA1c decrease of <1%, 73.9% were able to stop insulin and 26.1% were able to reduce. Even in those whose HbA1c increased up to 1%, 53.3% were still able to reduce or stop insulin. Only three participants had their A1c rise > 1% after insulin cessation (Figure 1).

**Figure 2:**
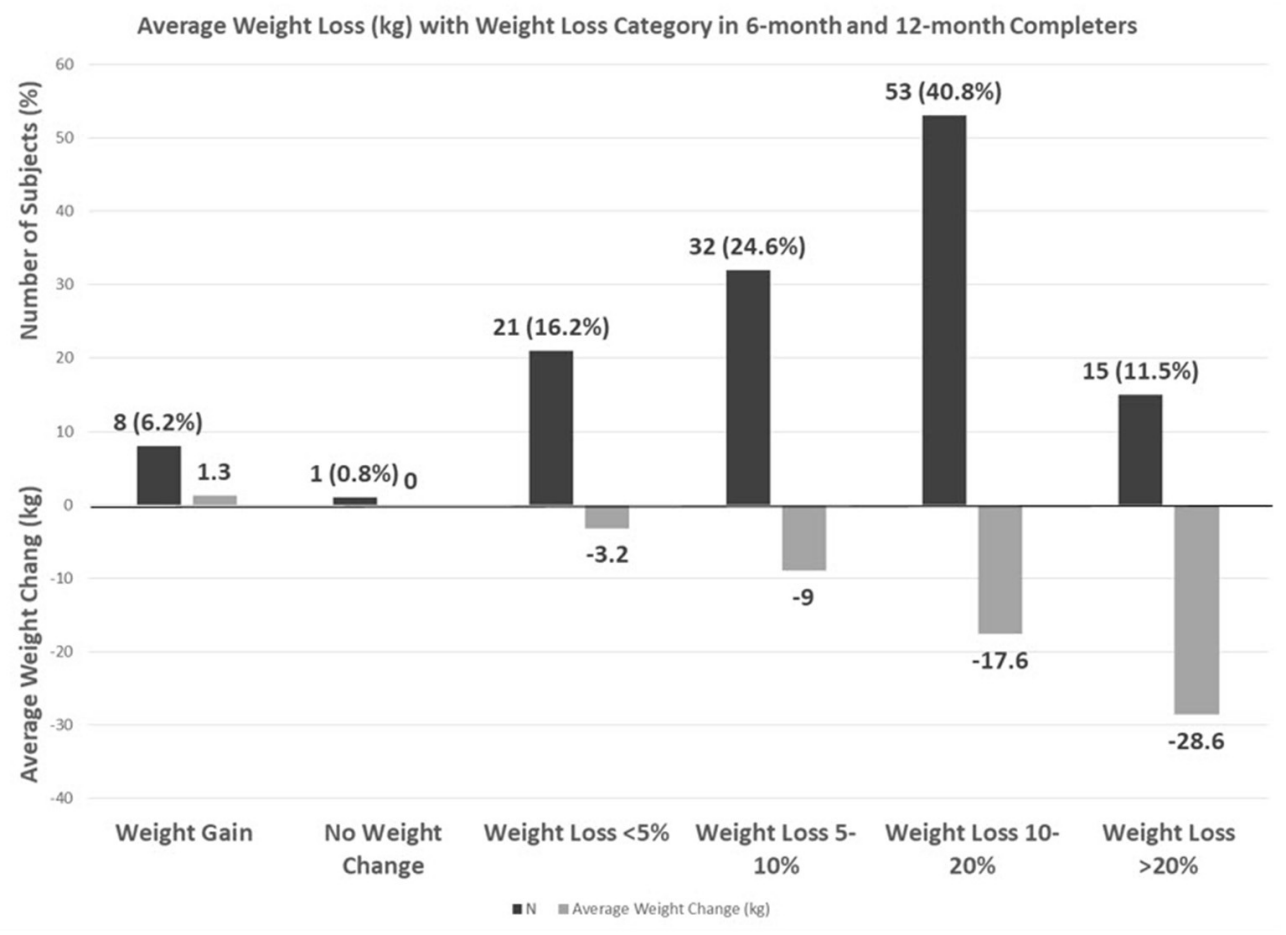
Average weight loss (kg) with weight loss category in 6-month and 12-month completers.

On combining 6 and 12-month completers, 24.6% had a 5–10% weight loss, 40.8% had a 10–20% weight loss, and 11.5% had a > 20% weight loss with an average loss of 28.6 kg (Figure 2).

## Discussion

This study, conducted in a real-world clinical setting, clearly demonstrates that an LCD can have a profound impact on the treatment of T2DM. Despite initially being managed with standard of care using current T2DM guidelines, including the use of insulin therapy, this cohort had suboptimal control of their diabetes and Class 3 obesity before starting the LCD intervention. The LCD enabled the participants to achieve control of their T2DM while simultaneously reducing or stopping their insulin consumption and losing weight. This intervention was more successful than any other medication for T2DM, and the weight loss was also greater than, even that of, intragastric balloon procedures (14).

This is an incredibly important finding given the rise in cases and financial implications of T2DM on our society. Recently, 1 in 10 Americans have diabetes, with T2DM accounting for 90–95% of cases, and 1 in 3 Americans have prediabetes (1). In addition, T2DM is becoming increasingly prevalent among youth (age <20) in the US (3), with a 4.8% annual increase in incidence and projected doubling of T2DM in youth by 2050 (15). Diabetes consistently ranks as one of the top contributors to healthcare expenditure among chronic diseases, with a total cost of $327 billion in the US in 2017 (16), including direct medical costs and decreased productivity (16). Furthermore, diabetes costs of 67.3% in the US are provided by government insurance, including Medicare, Medicaid, and the military. Patients with diabetes individually spend an average of $16,752 annually on medical care, with $9,601 attributed to diabetes (16). This is 2.3 times higher than the medical expenditure of patients without diabetes (16).

For people who have T2DM, being on insulin can further increase the burden to the individual and society. Specifically, insulin use may cause physical, psychological, and financial harm to patients, which can be underestimated by providers. In comparison with metformin and sulfonylureas, insulin use has been shown to have more adverse outcomes in terms of diabetes-related complications, cardiovascular events, cancer, and all-cause mortality. This is assumed to be caused by weight gain, atherogenic, and mitogenic effects from insulin is a growth factor and increased oxidative stress from induced vasodilation of arteriolar nitric oxide pathways (17). In addition, annually, an estimated 100,000 emergency room visits and 30,000 hospitalizations are attributed to insulin, either due to hypoglycemia or errors in administration (18). From a psychological perspective, adherence to an insulin regimen may be difficult for patients with limited diabetes knowledge, negative self-perceptions, fear of side effects or self-injection, concern about lifestyle restrictions, such as when traveling, and with the social stigma associated with injecting in public. These internal and external factors that influence the attitudes of patients toward insulin therapy can produce a complex “Psychological Insulin Resistance,” (19) which ultimately can worsen diabetes. From a cost perspective, insulin is expensive. It was estimated that in 2012 insulin therapy cost about $6 billion in the US. Those uninsured or in the coverage gap (“donut hole”) can incur substantial financial hardship or insulin may even be cost-prohibitive and they tend to go without it. Therefore, insulin cessation would dramatically benefit the patient in terms of cost, the complexity of dosing, administration, and risks associated with insulin therapy (18).

Many providers and patients believe that once insulin is started, it is unlikely to ever be discontinued; however, this study refutes this notion as patients who were on insulin for more than 10 years or those on hundreds of units were able to significantly reduce or even stop insulin while following an LCD. Specifically, for those where duration was known, of the 12 participants who were on insulin for 10 or more years, 100% of them were able to reduce and 50% of them were able to completely stop. Of 51 participants who were on more than 100 units of insulin, 21 were able to stop (41.2%) and stay off for 6 or 12 months when they were in the program. Of the 21 participants who were able to stop, 8 were able to come off by 1 month and 2 of the 8 were on very large doses of insulin (250 and 300 units). Thus, there is still hope for every patient with T2DM on insulin, and they should be made aware of this possibility. In addition, pause should also be taken into account prior to initiating insulin. Current ADA diabetes recommendations state that patients with A1c values ≥10% should be started on insulin (20). Although this study looked at taking participants off of insulin, the magnitude of benefit seen should encourage a trial of an LCD prior to starting insulin in willing patients. Since most of them stopped insulin within the first few months of starting an LCD, it can be a motivating factor to continue the lifestyle change. Despite the rapidly changing insulin requirements, close monitoring prevented any cases of symptomatic hypoglycemia. Participants were followed daily until insulin was discontinued or at a stable dose. None of the patients reported any other complications that required a visit to a provider, emergency room, or hospital admission.

In addition to the improvement in T2DM, our participants also had a substantial weight loss. The remarkable weight loss of 17 kg in the 12-month completers is significantly more than what is reported in most randomized control trials. A major dietary trial, the A to Z Weight Loss Study, comparing the Atkins, LEARN, Ornish, and Zone diets showed only a 1.6–4.7 kg weight loss (21).

It should be noted that in the case of 12-month completers, 13 were taking a GLP-1 on entry into the program and 27 (35.3%) were started at some point during the year. Of the 27 where a GLP-1 was initiated, 20 were able to stop insulin, 10 of which were able to stop insulin by 3 months. Of the 10, only 1 had started a GLP-1 prior to stopping insulin. GLP-1 agonists can add about a 1–1.5% HbA1c reduction and a 3.3 kg weight loss (22), but our results exceed these values, thus this effect was not solely due to the addition of the GLP-1.

The use of an LCD to treat T2DM is not a new concept, and there are many published trials. Indeed, our real-world study compared favorably to a 1-year study published in 2018 by Hallberg et al., where participants were placed on an LCKD with remote daily coaching and monitoring to ensure ketosis, and participants were compared with a control group following standard advice (23). The current study showed that 12-month completers had a 13.8% HbA1c reduction, with 97.6% reducing or discontinuing insulin and achieving a 17 kg weight loss, while the Hallberg study showed a 17.2% HbA1c reduction, 13.8 kg weight loss. They also reported that the control arm following conventional treatment showed a 2.6% increase in HbA1c at 1 year, highlighting the ineffectiveness of current guidelines (23). The current study population had similar demographic,s to the Hallberg study, with respect to age, sex, and starting weight; however, it also had some notable differences. Compared to their study with 7% black participants, this study had nearly an order of magnitude greater at 63%. In addition, their participants were continually monitored and counseled to try to achieve ketosis, while this study did not measure for ketones and likely included a subset of participants who were not in ketosis. As with the Hallberg study, the current study also included daily monitoring, but only until the insulin dose and blood glucose readings had stabilized. Further, since there are no restrictions on who can join the program, this study included complicated participants, many of whom may have been excluded from the Hallberg study, such as those with solid organ transplants (liver, kidney, and heart), cirrhosis, heart failure, HIV, pulmonary hypertension, end-stage renal disease on dialysis, and prior weight loss surgery.

There are many documented benefits of an LCD. Studies show improvement in all five features of the metabolic syndrome (24–26) as well as increased satiety, which can lead to lower total calorie consumption and weight loss (27). With regard to an LCKD, ketones, such as beta-hydroxybutyrate, have been theorized to have strong anti-oxidative and anti-inflammatory properties (28). This is particularly important as diabetes is an inflammatory state associated with increased levels of cytokines and immune activity that results in the classic downstream consequence of beta-cell “burnout” (29).

Despite the mounting evidence of the benefits of an LCD and the 2019 consensus report of the American Diabetes Association (ADA) including the LCD among the many eating patterns that can benefit patients with T2DM and highlighting that it has the most evidence for reducing glycemia (30), physicians and patients are still wary of its use, often citing the higher fat as a cause for concern; however, evidence is shifting away from demonizing fats as atherogenic. Indeed, in 2020, the Journal of the American College of Cardiology stated, “There is no robust evidence that current population-wide arbitrary upper limits on saturated fat consumption in the United States will prevent cardiovascular disease (CVD) or reduce mortality.” With regard to concern about saturated fat increasing low-density lipoprotein (LDL) cholesterol, they also stated that even in the minority who do have an increase in LDL, in most it is not due to increasing levels of small, dense LDL particles but rather larger LDL particles, which are much less strongly related to the risk of CVD (31). Other studies have shown that carbohydrates, specifically sugar, pose a significant ability to raise total cholesterol, LDL, and triglycerides while also lowering high-density lipoprotein (HDL) cholesterol, thereby increasing the cardiovascular risk (32).

This study brings up other interesting observations and questions for further evaluation. It is often thought that it is difficult to adhere to an LCD; however, nearly half of our participants completed 1 year in the program, and over two-thirds of participants completed 6-months or more. Many who dropped out at 3 and 6 months still had significant reductions in their insulin and HbA1c (30% dropped out before 3 months). Though some participants may have dropped out due to challenges in the feasibility or sustainability of an LCD, it is known that dietary interventions of all types are traditionally difficult to sustain. Though there is not much data on attrition rates in the obesity treatment literature, drop-out rates ranging from 10 to 80% have been cited (33). Thus, further exploration of drop-out rates in obesity treatment, and LCD intervention, specifically, is warranted.

Regarding depression, contingency table analyses for categorical variables showed that the distribution of LCD intervention completers varied as a function of depression. There was a statistically significant difference as depression was present in 24% of 6- and 12-month completers compared with 47% in those who completed LCD intervention for 3 months or less (*P* = 0.02, Pearson's chi-square test). This suggests that dropouts or continuation in the LCD intervention program were influenced by the presence of underlying depression. This merits further investigation as a factor for the successful implementation of an LCD program. Finally, evaluating the short and long-term cost savings from reducing or discontinuing insulin and improving diabetes control compared with the cost of attending an intensive lifestyle program should be undertaken.

## Limitations

Given the real-world nature of this study, certain limitations exist. The study was not matched with a control arm of patients following existing T2DM dietary recommendations. Subjective dietary adherence was not obtained during the first 4 years of the program. Therefore, it was not captured for the early participants. Therefore, in the 12-month completers, adherence was only captured for 53/85 (62.4%). Of these 53, 69.8% reported dietary adherence of ≥ 75%. Given the successful outcomes of this study, it is conceivable though, and assumed, that a majority of the participants had good adherence. Without testing for ketones or calorie/macronutrient counting, there are no objective measures to capture diet compliance, and we instead rely on laboratory and weight improvements to indicate adherence. In addition, exercise is encouraged throughout the program as part of an overall healthy lifestyle but is not specifically prescribed as part of the weight loss plan. Thus, activity level and exercise frequency may have varied among participants, potentially impacting weight loss and metabolic variability. As mentioned above, the addition of a GLP-1 has also been a confounding variable.

## Conclusion

This study demonstrated that an LCD is an effective dietary intervention that can improve glycemic control, facilitate insulin reduction or cessation, and achieve weight loss. We believe that these results showing 70% of participants who completed 12-months were able to discontinue insulin will give immense confidence to patients and providers that discontinuing insulin is possible. With an increase in cases, the staggering impact that T2DM has on the individual and society, and the fact that current guidelines have been ineffective in slowing or reversing this trend, new options must be explored. An LCD should be discussed with every patient with T2DM, especially those on insulin, and a referral to an obesity medicine specialist should be made if the provider is not comfortable managing the dietary intervention. Though not without its limitations, this study prompts clinicians to question the current paradigm of diabetes management by offering a dietary treatment that not only addresses weight and hyperglycemia but also the overall health and wellness of patients.

## Data Availability Statement

The datasets presented in this article are not readily available because data is being analyzed for future manuscripts. Requests to access the datasets should be directed to Susan Wolver, MD at swolver@vcu.edu.

## Ethics Statement

Ethical review and approval was not required for the study on human participants in accordance with the local legislation and institutional requirements. Written informed consent for participation was not required for this study in accordance with the national legislation and the institutional requirements.

## Author Contributions

SW, KF, and EF: designed Redcap for study, data entry, contributor to manuscript. KF, ZA, BO, NC, T-MC, SW, and DS: data entry and contributor to manuscript. SJ, AJ, and PP: statistical analysis and contributor to manuscript. SW and PP: study concept and design. All authors contributed to the article and approved the submitted version.

## Conflict of Interest

The authors declare that the research was conducted in the absence of any commercial or financial relationships that could be construed as a potential conflict of interest.

## Publisher's Note

All claims expressed in this article are solely those of the authors and do not necessarily represent those of their affiliated organizations, or those of the publisher, the editors and the reviewers. Any product that may be evaluated in this article, or claim that may be made by its manufacturer, is not guaranteed or endorsed by the publisher.
