# Supplementary figures and images for: Clinical Use of a Real-World Low Carbohydrate Diet Resulting in Reduction of Insulin Dose, Hemoglobin A1c, and Weight
Source: Front Nutr. 2021 Aug 11;8:690855. doi: 10.3389/fnut.2021.690855 (PMC8385129; doi:10.3389/fnut.2021.690855)

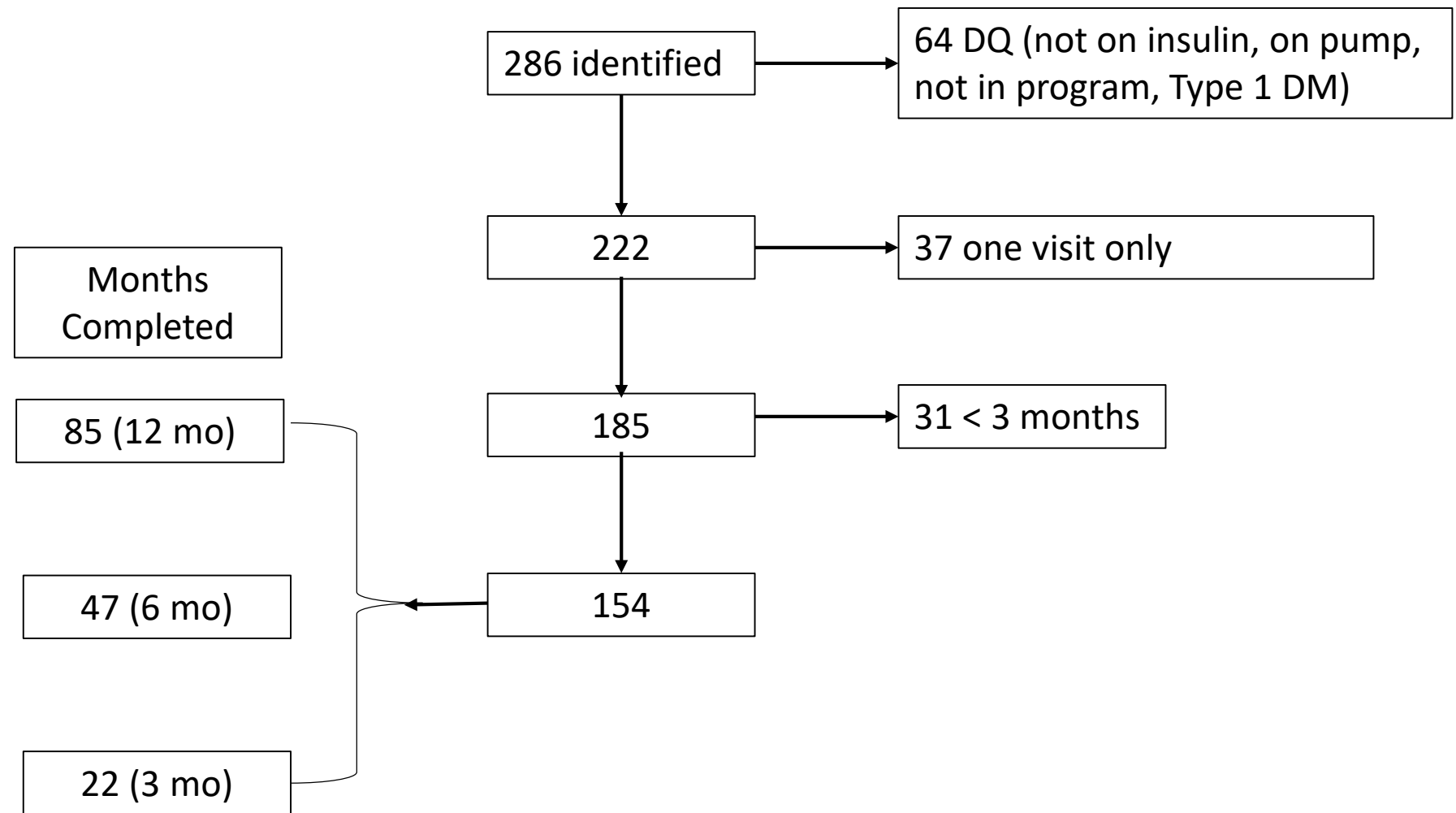

Supplement: Supplementary file 1 [file Data_Sheet_1.PDF]
